# Supplementary material for: Improved transformation efficiency in Mycoplasma hominis enables disruption of the MIB–MIP system targeting human immunoglobulins
Source: Microbiol Spectr. 2023 Sep 22;11(5):e01873-23. doi: 10.1128/spectrum.01873-23 (PMC10581049; doi:10.1128/spectrum.01873-23)
Supplement: Figure S1 — In silico analysis of the putative regulatory regions. [file spectrum.01873-23-s0001.docx]

**Figure S1. *In silico* analysis of the putative regulatory regions**

**A.** **Intergenic region between the locus MHO_0510 coding for a conserved hypothetical protein (+) and the locus MHO_0520 coding for the elongation factor Tu (+): 573 bp** (NC_013511.1; position 65736-66310)

5’-3’

AAAAATAAAAATTTTGAAATTATTAAAAAAGTATGGTAAAATGTATTTCGCAATTTGAAAAAAGCGATCGTTAAATTGCTCAAATTGCCGCTTAAAAAAATAAATATTATAGTTATATAATATACTCTCGCTCTTTTCTTATATTTATAATAAACCCTTATTTATTTTATACACATATACATACAACCCTTATTTTTTATATTTCCTTAACTTAGAACTTGAAGTTCAACTTATTATTAAATTAAATAATCAAAAGCTTATATTGTTCGATTTTTAGCGAGAGTAATCTTATTTTAAAAAATATTTCTTTCAAAATAGTAAACAAAAATGTTTGCTATTTTTTAATATTTTTGTTTAAAATATATAAGTACATAAATATCACTATTTTATGGCCAAAATTAAATCATTTACATTAAAAAAATATTTTTTTTGTTAAAATTACATTGCTATAAATGCGCGCTTTTGGACAGGACATAGTTGAAAGGCTATGCTAAGGTAGGTTAAACGCGTTTAATCAAAAGATTTAATAAATATTAAAAAAATTAAAATGTAATTTATTGAAAGGAAATTATTATG

**B.** **Intergenic region between the locus MHO_0680 coding for a hypothetical protein (-) and the locus MHO_0690 coding for the arginine deiminase (+): 448 bp** (NC_013511.1; position 88340-88789)

5’-3’

Sense strand

AATCACCTCATAAAATTTGTTTCTTATTATAACACTTTCTTACAATATATCTAATAAGCAATTCTCTATATATTATATAGATATAACTTTCATAATAACAAAAAATGTACATAATGGCGAAATTTTAGTTAACTATAGTTAGATTTTTTTAAGCAAGTATATTTTATTTACTATAAACAGTTAAAATATTTTGAACTTTGTTTCAAATTCAAATTATAAAAATAAAGATTATTTTATGCAGAATCTTTATATATTTTAACTTTTTTAACATTAGCCTGCACTAATTTTAAAATATGATGCATTTTATGCATAAAAAATGTTTTAAATTCTTTGCAAATTTATAAACAAAAAAATATAGTATAATTTATTACGTATAAATCATAATTAATGCAATAAAAGTGCATTTGTATTTATACGCTATAACATATATATTTGAAAGGAATAAAAAATG

Reverse complementary strand TTTTTATTCCTTTCAAATATATATGTTATAGCGTATAAATACAAATGCACTTTTATTGCATTAATTATGATTTATACGTAATAAATTATACTATATTTTTTTGTTTATAAATTTGCAAAGAATTTAAAACATTTTTTATGCATAAAATGCATCATATTTTAAAATTAGTGCAGGCTAATGTTAAAAAAGTTAAAATATATAAAGATTCTGCATAAAATAATCTTTATTTTTATAATTTGAATTTGAAACAAAGTTCAAAATATTTTAACTGTTTATAGTAAATAAAATATACTTGCTTAAAAAAATCTAACTATAGTTAACTAAAATTTCGCCATTATGTACATTTTTTGTTATTATGAAAGTTATATCTATATAATATATAGAGAATTGCTTATTAGATATATTGTAAGAAAGTGTTATAATAAGAAACAAATTTTATGAGGTGATTATG

**C. Synthetic regulatory region named SynMyco** (Montero-Blay *et* *al*, 2019, DNA Research (16))

TTATAAGCCTCTCTACTGCAATTTATCTCAACTTTGATATAATTAAAGACATACGAAAGGATTTTAATATG

**D. Multiple scheme alignment of RR composition according to the consensus composition (TGC)-Xn-(TAWAAT)-Xn- (AAAGGA)-Xn-(ATG)**

....|....| ....|....| ....|....| ....|....| ....|....| ....|....| ....|....|

10 20 30 40 50 60 70

**1.**  CATAGTTGAA AGGCTATGCT AAGGTAGGTT AAACGCGTTT AATCAAAAGA TTTAATAAAT ATTAAAAAAA

**2.**  AATGTTTTAA ATTCTTTGCA AATTTATAAA CAAAAAAATA TAGTATAATT TATTACGTAT AAATCATAAT

**3.**  TTATAAGCCT CTCTACTGCA ATTTATCTCA ACTTTGATAT AATTAAAGAC ATACGAAAGG ATTTTAATAT

....|....| ....|....| ....|....| ....|....| ....|....| ....|....| ....|.

80 90 100 110 120 130

**1.**  TTAAAATGTA ATTTATTGAA AGGAAATTAT TATG...... .......... .......... ......

**2.**  TAATGCAATA AAAGTGCATT TGTATTTATA CGCTATAACA TATATATTTG AAAGGAATAA AAAATG

**3.**  G......... .......... .......... .......... .......... .......... ......

**A. and B.** Transcription and translation determinants were sought in DNA sequences located upstream of the elongation factor Tu (MHO_0520) and the arginine deiminase (MHO_0690) coding regions on the basis of the genomic sequence of the *M. hominis* reference strain PG21 (NC_013511.1), (Pereyre *et al*, 2009, PLoS Genet (10)). The orientation of coding DNA sequences is specified into parenthesis. The four main domains are highlighted in yellow for the putative -35 box, in blue for the putative pribnow box, in green for the putative ribosomal binding site (RBS) sequence and in red for the translation starting codon. The DNA sequence selected as the putative regulatory region in this study is underlined. **B.** Putative regulatory domains of the locus MHO_0680 are highlighted in the reverse complementary strand. **C.** The synthetic regulatory region named SynMyco is presented for comparison. **D.** Multiple scheme alignment of the RR composition of the regions upstream of the Tu (1) and arginine deiminase genes (2), and of the SynMyco RR (3).
