# Supplementary material for: Improved transformation efficiency in Mycoplasma hominis enables disruption of the MIB–MIP system targeting human immunoglobulins
Source: Microbiol Spectr. 2023 Sep 22;11(5):e01873-23. doi: 10.1128/spectrum.01873-23 (PMC10581049; doi:10.1128/spectrum.01873-23)
Supplement: Figure S2 — Multiple sequence alignment of tetracycline resistance variants. [file spectrum.01873-23-s0002.docx]

**Figure S2. Multiple sequence alignment of tetracycline resistance variants.**

**a.** Alignment of *tet*(M) sequences of the pMT85-Tet plasmid and seven *M*. *hominis* (*Mho*) clinical isolates resistant to tetracycline (5860, 6892, 6227, 5571, 6585 (Meygret *et al*, 2018, J Antimicrob Chemother (22)), Sprott [accession number CP011538.1], and 2539 [accession number NZ_CP026341.1]). The consensus nucleotide sequence corresponding to that of the pMT85-Tet plasmid (*Enterococcus*-derived) is indicated above and indication of single nucleotide substitutions is provided below (n=30).

....|....| ....|....| ....|....| ....|....| ....|....|

5 15 25 35 45

*tet*(M) pMT85-Tet .......... .....ATGAA AATTATTAAT ATTGGAGTTT TAGCTCATGT

*tet*(M) *Mho* 5860 .......... .....~~~~~ ~~~~~~~~~~ ~~~~~~~~~~ ~~~~~~~~~~

*tet*(M) *Mho* 6892 .......... .....~~~~~ ~~~~~~~~~~ ~~~~~~~~~~ ~~~~~~~~~~

*tet*(M) *Mho* 6227 .......... .....~~~~~ ~~~~~~~~~~ ~~~~~~~~~~ ~~~~~~~~~~

*tet*(M) *Mho* 5571 .......... .....~~~~~ ~~~~~~~~~~ ~~~~~~~~~~ ~~~~~~~~~~

*tet*(M) *Mho* 6585 .......... .....~~~~~ ~~~~~~~~~~ ~~~~~~~~~~ ~~~~~~~~~~

*tet*(M) *Mho* Sprott .......... .....~~~~~ ~~~~~~~~~~ ~~~~~~~~~~ ~~~~~~~~~~

*tet*(M) *Mho* 2539 .......... .....~~~~~ ~~~~~~~~~~ ~~~~~~~~~~ ~~~~~~~~~~

....|....| ....|....| ....|....| ....|....| ....|....|

55 65 75 85 95

*tet*(M) pMT85-Tet TGATGCAGGA AAAACTACCT TAACAGAAAG CTTATTATAT AACAGTGGAG

*tet*(M) *Mho* 5860 ~~~~~~~~~~ ~~~~~~~~~~ ~~~~~~~~~~ ~~~~~~~~~~ ~~~~~~~~~~

*tet*(M) *Mho* 6892 ~~~~~~~~~~ ~~~~~~~~~~ ~~~~~~~~~~ ~~~~~~~~~~ ~~~~~~~~~~

*tet*(M) *Mho* 6227 ~~~~~~~~~~ ~~~~~~~~~~ ~~~~~~~~~~ ~~~~~~~~~~ ~~~~~~~~~~

*tet*(M) *Mho* 5571 ~~~~~~~~~~ ~~~~~~~~~~ ~~~~~~~~~~ ~~~~~~~~~~ ~~~~~~~~~~

*tet*(M) *Mho* 6585 ~~~~~~~~~~ ~~~~~~~~~~ ~~~~~~~~~~ ~~~~~~~~~~ ~~~~~~~~~~

*tet*(M) *Mho* Sprott ~~~~~~~~~~ ~~~~~~~~~~ ~~~~~~~~~~ ~~~~~~~~~~ ~~~~~~~~~~

*tet*(M) *Mho* 2539 ~~~~~~~~~~ ~~~~~~~~~~ ~~~~~~~~~~ ~~~~~~~~~~ ~~~~~~~~~~

....|....| ....|....| ....|....| ....|....| ....|....|

105 115 125 135 145

*tet*(M) pMT85-Tet CGATTACAGA ATTAGGAAGC GTGGACAAAG GTACAACGAG GACGGATAAT

*tet*(M) *Mho* 5860 ~~~~~~~~~~ ~~~~~~~~~~ ~~~~~~~~~~ ~~~~~~~~~~ ~~~~~~~~~~

*tet*(M) *Mho* 6892 ~~~~~~~~~~ ~~~~~~~~~~ ~~~~~~~~~~ ~~~~~~~~~~ ~~~~~~~~~~

*tet*(M) *Mho* 6227 ~~~~~~~~~~ ~~~~~~~~~~ ~~~~~~~~~~ ~~~~~~~~~~ ~~~~~~~~~~

*tet*(M) *Mho* 5571 ~~~~~~~~~~ ~~~~~~~~~~ ~~~~~~~~~~ ~~~~~~~~~~ ~~~~~~~~~~

*tet*(M) *Mho* 6585 ~~~~~~~~~~ ~~~~~~~~~~ ~~~~~~~~~~ ~~~~~~~~~~ ~~~~~~~~~~

*tet*(M) *Mho* Sprott ~~~~~~~~~~ ~~~~~~~~~~ ~~~~~~~~~~ ~~~~~~~~~~ ~~~~~~~~~~

*tet*(M) *Mho* 2539 ~~~~~~~~~~ ~~~~~~~~~~ ~~~~~~~~~~ ~~~~~~~~~~ ~~~~~~~~~~

....|....| ....|....| ....|....| ....|....| ....|....|

155 165 175 185 195

*tet*(M) pMT85-Tet ACGCTTTTAG AACGTCAGAG AGGAATTACA ATTCAGACAG GAATAACCTC

*tet*(M) *Mho* 5860 ~~~~~~~~~~ ~~~~~~~~~~ ~~~~~~~~~~ ~~~~~~~~~~ ~~~~~~~~~~

*tet*(M) *Mho* 6892 ~~~~~~~~~~ ~~~~~~~~~~ ~~~~~~~~~~ ~~~~~A~~~~ ~~~~~~~~~~

*tet*(M) *Mho* 6227 ~~~~~~~~~~ ~~~~~~~~~~ ~~~~~~~~~~ ~~~~~~~~~~ ~~~~~~~~~~

*tet*(M) *Mho* 5571 ~~~~~~~~~~ ~~~~~~~~~~ ~~~~~~~~~~ ~~~~~~~~~~ ~~~~~~~~~~

*tet*(M) *Mho* 6585 ~~~~~~~~~~ ~~~~~~~~~~ ~~~~~~~~~~ ~~~~~~~~~~ ~~~~~~~~~~

*tet*(M) *Mho* Sprott ~~~~~~~~~~ ~~~~~~~~~~ ~~~~~~~~~~ ~~~~~~~~~~ ~~~~~~~~~~

*tet*(M) *Mho* 2539 ~~~~~~~~~~ ~~~~~~~~~~ ~~~~~~~~~~ ~~~~~~~~~~ ~~~~~~~~~~

....|....| ....|....| ....|....| ....|....| ....|....|

205 215 225 235 245

*tet*(M) pMT85-Tet TTTTCAGTGG GAAAATACGA AGGTGAACAT CATAGACACG CCAGGACATA

*tet*(M) *Mho* 5860 ~~~~~~~~~~ ~~~~~~~~~~ ~~~~~~~~~~ ~~~~~~~~~~ ~~~~~~~~~~

*tet*(M) *Mho* 6892 ~~~~~~~~~~ ~~~~~~~~~~ ~~~~~~~~~~ ~~~~~~~~~~ ~~~~~~~~~~

*tet*(M) *Mho* 6227 ~~~~~~~~~~ ~~~~~~~~~~ ~~~~~~~~~~ ~~~~~~~~~~ ~~~~~~~~~~

*tet*(M) *Mho* 5571 ~~~~~~~~~~ ~~~~~~~~~~ ~~~~~~~~~~ ~~~~~~~~~~ ~~~~~~~~~~

*tet*(M) *Mho* 6585 ~~~~~~~~~~ ~~~~~~~~~~ ~~~~~~~~~~ ~~~~~~~~~~ ~~~~~~~~~~

*tet*(M) *Mho* Sprott ~~~~~~~~~~ ~~~~~~~~~~ ~~~~~~~~~~ ~~~~~~~~~~ ~~~~~~~~~~

*tet*(M) *Mho* 2539 ~~~~~~~~~~ ~~~~~~~~~~ ~~~~~~~~~~ ~~~~~~~~~~ ~~~~~~~~~~

....|....| ....|....| ....|....| ....|....| ....|....|

255 265 275 285 295

*tet*(M) pMT85-Tet TGGATTTCTT AGCAGAAGTA TATCGTTCAT TATCAGTTTT AGATGGGGCA

*tet*(M) *Mho* 5860 ~~~~~~~~~~ ~~~~~~~~~~ ~~~~~~~~~~ ~~~~~~~~~~ ~~~~~~~~~~

*tet*(M) *Mho* 6892 ~~~~~~~~~~ ~~~~~~~~~~ ~~~~~~~~~~ ~~~~~~~~~~ ~~~~~~~~~~

*tet*(M) *Mho* 6227 ~~~~~~~~~~ ~~~~~~~~~~ ~~~~~~~~~~ ~~~~~~~~~~ ~~~~~~~~~~

*tet*(M) *Mho* 5571 ~~~~~~~~~~ ~~~~~~~~~~ ~~~~~~~~~~ ~~~~~~~~~~ ~~~~~~~~~~

*tet*(M) *Mho* 6585 ~~~~~~~~~~ ~~~~~~~~~~ ~~~~~~~~~~ ~~~~~~~~~~ ~~~~~~~~~~

*tet*(M) *Mho* Sprott ~~~~~~~~~~ ~~~~~~~~~~ ~~~~~~~~~~ ~~~~~~~~~~ ~~~~~~~~~~

*tet*(M) *Mho* 2539 ~~~~~~~~~~ ~~~~~~~~~~ ~~~~~~~~~~ ~~~~~~~~~~ ~~~~~~~~~~

....|....| ....|....| ....|....| ....|....| ....|....|

305 315 325 335 345

*tet*(M) pMT85-Tet ATTCTACTGA TTTCTGCAAA AGATGGCGTA CAAGCACAAA CTCGTATATT

*tet*(M) *Mho* 5860 ~~~~~~~~~~ ~~~~~~~~~~ ~~~~~~~~~~ ~~~~~~~~~~ ~~~~~~~~~~

*tet*(M) *Mho* 6892 ~~~~~~~~~~ ~~~~~~~~~~ ~~~~~~~~~~ ~~~~~~~~~~ ~~~~~~~~~~

*tet*(M) *Mho* 6227 ~~~~~~~~~~ ~~~~~~~~~~ ~~~~~~~~~~ ~~~~~~~~~~ ~~~~~~~~~~

*tet*(M) *Mho* 5571 ~~~~~~~~~~ ~~~~~~~~~~ ~~~~~~~~~~ ~~~~~~~~~~ ~~~~~~~~~~

*tet*(M) *Mho* 6585 ~~~~~~~~~~ ~~~~~~~~~~ ~~~~~~~~~~ ~~~~~~~~~~ ~~~~~~~~~~

*tet*(M) *Mho* Sprott ~~~~~~~~~~ ~~~~~~~~~~ ~~~~~~~~~~ ~~~~~~~~~~ ~~~~~~~~~~

*tet*(M) *Mho* 2539 ~~~~~~~~~~ ~~~~~~~~~~ ~~~~~~~~~~ ~~~~~~~~G~ ~~~~~~~~~~

....|....| ....|....| ....|....| ....|....| ....|....|

355 365 375 385 395

*tet*(M) pMT85-Tet ATTTCATGCA CTTAGGAAAA TGGGGATTCC CACAATCTTT TTTATCAATA

*tet*(M) *Mho* 5860 ~~~~~~~~~~ ~~~~~~~~~~ ~~~~~~~~~~ ~~~~~~~~~~ ~~~~~~~~~~

*tet*(M) *Mho* 6892 ~~~~~~~~~~ ~~~~~~~~~~ ~~~~~~~~~~ ~~~~~~~~~~ ~~~~~~~~~~

*tet*(M) *Mho* 6227 ~~~~~~~~~~ ~~~~~~~~~~ ~~~~~~~~~~ ~~~~~~~~~~ ~~~~~~~~~~

*tet*(M) *Mho* 5571 ~~~~~~~~~~ ~~~~~~~~~~ ~~~~~~~~~~ ~~~~~~~~~~ ~~~~~~~~~~

*tet*(M) *Mho* 6585 ~~~~~~~~~~ ~~~~~~~~~~ ~~~~~~~~~~ ~~~~~~~~~~ ~~~~~~~~~~

*tet*(M) *Mho* Sprott ~~~~~~~~~~ ~~~~~~~~~~ ~~~~~~~~~~ ~~~~~~~~~~ ~~~~~~~~~~

*tet*(M) *Mho* 2539 ~~~~~~~~~~ ~~~~~~~~~~ ~~~~~~~~~~ ~~~~~~~~~~ ~~~~~~~~~~

....|....| ....|....| ....|....| ....|....| ....|....|

405 415 425 435 445

*tet*(M) pMT85-Tet AGATTGACCA AAATGGAATT GATTTATCAA CGGTTTATCA GGATATTAAA

*tet*(M) *Mho* 5860 ~~~~~~~~~~ ~~~~~~~~~~ ~~~~~~~~~~ ~~~~~~~~~~ ~~~~~~~~~~

*tet*(M) *Mho* 6892 ~~~~~~~~~~ ~~~~~~~~~~ ~~~~~~~~~~ ~~~~~~~~~~ ~~~~~~~~~~

*tet*(M) *Mho* 6227 ~~~~~~~~~~ ~~~~~~~~~~ ~~~~~~~~~~ ~~~~~~~~~~ ~~~~~~~~~~

*tet*(M) *Mho* 5571 ~~~~~~~~~~ ~~~~~~~~~~ ~~~~~~~~~~ ~~~~~~~~~~ ~~~~~~~~~~

*tet*(M) *Mho* 6585 ~~~~~~~~~~ ~~~~~~~~~~ ~~~~~~~~~~ ~~~~~~~~~~ ~~~~~~~~~~

*tet*(M) *Mho* Sprott ~~~~~~~~~~ ~~~~~~~~~~ ~~~~~~~~~~ ~~~~~~~~~~ ~~~~~~~~~~

*tet*(M) *Mho* 2539 ~~~~~~~~~~ ~~~~~~~~~~ ~~~~~~~~~~ ~~~~~~~~~~ ~~~~~~~~~~

....|....| ....|....| ....|....| ....|....| ....|....|

455 465 475 485 495

*tet*(M) pMT85-Tet GAGAAACTTT CTGCCGAAAT TGTAATCAAA CAGAAGGTAG AACTGTATCC

*tet*(M) *Mho* 5860 ~~~~~~~~~~ ~~~~~~~~~~ ~~~~~~~~~~ ~~~~~~~~~~ ~~~~~~~~~~

*tet*(M) *Mho* 6892 ~~~~~~~~~~ ~~~~~~~~~~ ~~~~~~~~~~ ~~~~~~~~~~ ~~~~~~~~~~

*tet*(M) *Mho* 6227 ~~~~~~~~~~ ~~~~~~~~~~ ~~~~~~~~~~ ~~~~~~~~~~ ~~~~~~~~~~

*tet*(M) *Mho* 5571 ~~~~~~~~~~ ~~~~~~~~~~ ~~~~~~~~~~ ~~~~~~~~~~ ~~~~~~~~~~

*tet*(M) *Mho* 6585 ~~~~~~~~~~ ~~~~~~~~~~ ~~~~~~~~~~ ~~~~~~~~~~ ~~~~~~~~~~

*tet*(M) *Mho* Sprott ~~~~~~~~~~ ~~~~~~~~~~ ~~~~~~~~~~ ~~~~~~~~~~ ~~~~~~~~~~

*tet*(M) *Mho* 2539 ~~~~~~~~~~ ~~~~~~~~~~ ~~~~~~~~~~ ~~~~~~~~~~ ~~~~~~~~~~

....|....| ....|....| ....|....| ....|....| ....|....|

505 515 525 535 545

*tet*(M) pMT85-Tet TAATATGTGT GTGACGAACT TTACCGAATC TGAACAATGG GATACGGTAA

*tet*(M) *Mho* 5860 ~~~~~~~~~~ ~~~~~~~~~~ ~~~~~~~~~~ ~~~~~~~~~~ ~~~~~~~~~~

*tet*(M) *Mho* 6892 ~~~~~~~~~~ ~~~~~~~~~~ ~~~~~~~~~~ ~~~~~~~~~~ ~~~~~~~~~~

*tet*(M) *Mho* 6227 ~~~~~~~~~~ ~~~~~~~~~~ ~~~~~~~~~~ ~~~~~~~~~~ ~~~~~~~~~~

*tet*(M) *Mho* 5571 ~~~~~~~~~~ ~~~~~~~~~~ ~~~~~~~~~~ ~~~~~~~~~~ ~~~~~~~~~~

*tet*(M) *Mho* 6585 ~~~~~~~~~~ ~~~~~~~~~~ ~~~~~~~~~~ ~~~~~~~~~~ ~~~~~~~~~~

*tet*(M) *Mho* Sprott ~~~~~~~~~~ ~~~~~~~~~~ ~~~~~~~~~~ ~~~~~~~~~~ ~~~~~~~~~~

*tet*(M) *Mho* 2539 ~~~~~~~~~~ ~~~~~~~~~~ ~~~~~~~~~~ ~~~~~~~~~~ ~~~~~~~~~~

....|....| ....|....| ....|....| ....|....| ....|....|

555 565 575 585 595

*tet*(M) pMT85-Tet TAGAGGGAAA CGATGACCTT TTAGAGAAAT ATATGTCCGG TAAATCATTA

*tet*(M) *Mho* 5860 ~~~~~~~~~~ ~~~~~~~~~~ ~~~~~~~~~~ ~~T~~~~~~~ ~~~~~~~~~~

*tet*(M) *Mho* 6892 ~~~~~~~~~~ ~~~~~~~~~~ ~~~~~~~~~~ ~~~~~~~~~~ ~~~~~~~~~~

*tet*(M) *Mho* 6227 ~~~~~~~~~~ ~~~~~~~~~~ ~~~~~~~~~~ ~~~~~~~~~~ ~~~~~~~~~~

*tet*(M) *Mho* 5571 ~~~~~~~~~~ ~~~~~~~~~~ ~~~~~~~~~~ ~~~~~~~~~~ ~~~~~~~~~~

*tet*(M) *Mho* 6585 ~~~~~~~~~~ ~~~~~~~~~~ ~~~~~~~~~~ ~~~~~~~~~~ ~~~~~~~~~~

*tet*(M) *Mho* Sprott ~~~~~~~~~~ ~~~~~~~~~~ ~~~~~~~~~~ ~~~~~~~~~~ ~~~~~~~~~~

*tet*(M) *Mho* 2539 ~~~~~~~~~~ ~~~~~~~~~~ ~~~~~~~~~~ ~~~~~~~~~~ ~~~~~~~~~~

....|....| ....|....| ....|....| ....|....| ....|....|

605 615 625 635 645

*tet*(M) pMT85-Tet GAAGCATTGG AACTCGAACA AGAGGAAAGC ATAAGATTTC ATAATTGTTC

*tet*(M) *Mho* 5860 ~~~~~~~~~~ ~~~~~~~~~~ ~~~~~~G~~~ ~~~~~~~~~~ ~G~~~~~~~~

*tet*(M) *Mho* 6892 ~~~~~~~~~~ ~~~~~~~~~~ ~~~~~~G~~~ ~~~~~~~~~~ ~G~~~~~~~~

*tet*(M) *Mho* 6227 ~~~~~~~~~~ ~~~~~~~~~~ ~~~~~~~~~~ ~~~~~~~~~~ ~G~~~~~~~~

*tet*(M) *Mho* 5571 ~~~~~~~~~~ ~~~~~~~~~~ ~~~~~~~~~~ ~~~~~~~~~~ ~G~~~~~~~~

*tet*(M) *Mho* 6585 ~~~~~~~C~~ ~~~~~~~~~~ ~~~~~~~~~~ ~~~~~~~~~~ ~G~~~~~~~~

*tet*(M) *Mho* Sprott ~~~~~~~~~~ ~~~~~~~~~~ ~~~~~~~~~~ ~~~~~~~~~~ ~G~~~~~~~~

*tet*(M) *Mho* 2539 ~~~~~~~~~~ ~~~~~~~~~~ ~~~~~~~~~~ ~~~~~~~~~~ ~G~~~~~~~~

....|....| ....|....| ....|....| ....|....| ....|....|

655 665 675 685 695

*tet*(M) pMT85-Tet CCTGTTCCCT GTTTATCACG GAAGTGCAAA AAACAATATA GGGATTGATA

*tet*(M) *Mho* 5860 ~~~~~~~~~~ ~~~~~~~~~~ ~~~~~~~~~~ ~~~~~~~~~~ ~~~~~~~~~~

*tet*(M) *Mho* 6892 ~~~~~~~~~~ ~~~~~~~~~~ ~~~~~~~~~~ ~~~~~~~~~~ ~~~~~~~~~~

*tet*(M) *Mho* 6227 ~~~~~~~~~~ ~~~~~~~~~~ ~~~~~~~~~~ ~~~~~~~~~~ ~~~~~~~~~~

*tet*(M) *Mho* 5571 ~~~~~~~~~~ ~~~~~~~~~~ ~~~~~~~~~~ ~~~~~~~~~~ ~~~~~~~~~~

*tet*(M) *Mho* 6585 ~~~~~~~~~~ ~~~~~~~~~~ ~~~~~~~~~~ ~~~~~~~~~~ ~~~~~~~~~~

*tet*(M) *Mho* Sprott ~~~~~~~~~~ ~~~~~~~~~~ ~~~~~~~~~~ ~~~~~~~~~~ ~~~~~~~~~~

*tet*(M) *Mho* 2539 ~~~~~~~~~~ ~~~~~~~~~~ ~~~~~~~~~~ ~~~~~~~~~~ ~~~~~~~~~~

....|....| ....|....| ....|....| ....|....| ....|....|

705 715 725 735 745

*tet*(M) pMT85-Tet ACCTTATAGA AGTGATTACG AATAAATTTT ATTCATCAAC ACATCGAGGT

*tet*(M) *Mho* 5860 ~~~~~~~~~~ ~~~~~~~~~~ ~~~~~~~~~~ ~~~~~~~~~~ ~~~~~~~~~~

*tet*(M) *Mho* 6892 ~~~~~~~~~~ ~~~~~~~~~~ ~~~~~~~~~~ ~~~~~~~~~~ ~~~~~~~~~~

*tet*(M) *Mho* 6227 ~~~~~~~~~~ ~~~~~~~~~~ ~~~~~~~~~~ ~~~~~~~~~~ ~~~~~~~~~~

*tet*(M) *Mho* 5571 ~~~~~~~~~~ ~~~~~~~~~~ ~~~~~~~~~~ ~~~~~~~~~~ ~~~~~~~~~~

*tet*(M) *Mho* 6585 ~~~~~~~~~~ ~~~~~~~~~~ ~~~~~~~~~~ ~~~~~~~~~~ ~~~~~~~~~~

*tet*(M) *Mho* Sprott ~~~~~~~~~~ ~~~~~~~~~~ ~~~~~~~~~~ ~~~~~~~~~~ ~~~~~~~~~~

*tet*(M) *Mho* 2539 ~~~~~~~~~~ ~~~~~~~~~~ ~~~~~~~~~~ ~~~~~~~~~~ ~~~~~~~~~~

....|....| ....|....| ....|....| ....|....| ....|....|

755 765 775 785 795

*tet*(M) pMT85-Tet CCGTCTGAAC TTTGCGGAAA TGTTTTCAAA ATTGAATATA CAAAAAAAAG

*tet*(M) *Mho* 5860 ~A~~~~~~~~ ~~~~~~~~~~ A~~~~~~~~~ ~~~~~G~~~~ ~GG~~~~~~~

*tet*(M) *Mho* 6892 ~A~~~~~~~~ ~~~~~~~~~~ A~~~~~~~~~ ~~~~~G~~~~ ~GG~~~~~~~

*tet*(M) *Mho* 6227 ~A~~~~~~~~ ~~~~~~~~~~ A~~~~~~~~~ ~~~~~G~~~~ ~GG~~~~~~~

*tet*(M) *Mho* 5571 ~A~~~~~~~~ ~~~~~~~~~~ A~~~~~~~~~ ~~~~~G~~~~ ~GG~~~~~~~

*tet*(M) *Mho* 6585 ~A~~~~~~~~ ~~~~T~~~~~ A~~~~~~~~~ ~~~~~G~~~~ ~GG~~~~~~~

*tet*(M) *Mho* Sprott ~A~~~~~~~~ ~~~~~~~~~~ A~~~~~~~~~ ~~~~~G~~~~ ~GG~~~~~~~

*tet*(M) *Mho* 2539 ~A~~~~~~~~ ~~~~~~~~~~ A~~~~~~~~~ ~~~~~G~~~~ ~GG~~~~~~~

....|....| ....|....| ....|....| ....|....| ....|....|

805 815 825 835 845

*tet*(M) pMT85-Tet ACAACGTCTT GCATATATAC GCCTTTATAG TGGAGTACTA CATTTACGAG

*tet*(M) *Mho* 5860 ~~~G~~~~~~ ~~~~~~~~~~ ~T~~~~~~~~ ~~~C~~~~~G ~~~~~G~~~~

*tet*(M) *Mho* 6892 ~~~G~~~~~~ ~~~~~~~~~~ ~T~~~~~~~~ ~~~C~~~~~G ~~~~~G~~~~

*tet*(M) *Mho* 6227 ~~~G~~~~~~ ~~~~~~~~~~ ~T~~~~~~~~ ~~~C~~~~~G ~~~~~G~~~~

*tet*(M) *Mho* 5571 ~~~g~~~~~~ ~~~~~~~~~~ ~~~~~~~~~~ ~~~~~~~~~~ ~~~~~~~~~~

*tet*(M) *Mho* 6585 ~~~~~~~~~~ ~~~~~~~~~~ ~~~~~~~~~~ ~~~~~~~~~~ ~~~~~~~~~~

*tet*(M) *Mho* Sprott ~~~G~~~~~~ ~~~~~~~~~~ ~T~~~~~~~~ ~~~C~~~~~G ~~~~~G~~~~

*tet*(M) *Mho* 2539 ~~~G~~~~~~ ~~~~~~~~~~ ~T~~~~~~~~ ~~~C~~~~~G ~~~~~G~~~~

....|....| ....|....| ....|....| ....|....| ....|....|

855 865 875 885 895

*tet*(M) pMT85-Tet ATTCGGTTAG AGTATCAGAA AAAGAAAAAA TAAAAGTTAC AGAAATGTAT

*tet*(M) *Mho* 5860 ~~~~~~~~~~ ~A~~~~G~~~ ~~G~~~~~~~ ~~~~~A~~~~ ~~~~~~~~~~

*tet*(M) *Mho* 6892 ~~~~~~~~~~ ~A~~~~G~~~ ~~G~~~~~~~ ~~~~~A~~~~ ~~~~~~~~~~

*tet*(M) *Mho* 6227 ~~~T~~~~~~ ~A~~~~G~~~ ~~G~~~~~~~ ~~~~~A~~~~ ~~~~~~~~~~

*tet*(M) *Mho* 5571 ~~~~~~~~~~ ~A~~~~G~~~ ~~G~~~~~~~ ~~~~~A~~~T ~~~~~~~~~~

*tet*(M) *Mho* 6585 ~~~~~~~~~~ ~A~~~~G~~~ ~~G~~~~~~~ ~~~~~A~~~~ ~~~~~~~~~~

*tet*(M) *Mho* Sprott ~~~T~~~~~~ ~A~~~~G~~~ ~~G~~~~~~~ ~~~~~A~~~~ ~~~~~~~~~~

*tet*(M) *Mho* 2539 ~~~T~~~~~~ ~A~~~~G~~~ ~~G~~~~~~~ ~~~~~A~~~~ ~~~~~~~~~~

....|....| ....|....| ....|....| ....|....| ....|....|

905 915 925 935 945

*tet*(M) pMT85-Tet ACTTCAATAA ATGGTGAATT ATGTAAGATT GATAGAGCTT ATTCTGGAGA

*tet*(M) *Mho* 5860 ~~~~~~~~~~ ~~~~~~~~~~ ~~~~~~A~~C ~~~~AG~~~~ ~~~~~~~~~~

*tet*(M) *Mho* 6892 ~~~~~~~~~~ ~~~~~~~~~~ ~~~~~~A~~C ~~~~AG~~~~ ~~~~~~~~~~

*tet*(M) *Mho* 6227 ~~~~~~~~~~ ~~~~~~~~~~ ~~~~~~A~~C ~~~~AG~~~~ ~~~~~~~~~~

*tet*(M) *Mho* 5571 ~~~~~~~~~~ ~~~~~~~~~~ ~~~~~~A~~C ~~~~AG~~~~ ~~~~~~~~~~

*tet*(M) *Mho* 6585 ~~~~~~~~~~ ~~~~~~~~~~ ~~~~~~A~~C ~~~~AG~~~~ ~~~~~~~~~~

*tet*(M) *Mho* Sprott ~~~~~~~~~~ ~~~~~~~~~~ ~~~~~~A~~C ~~~~AG~~~~ ~~~~~~~~~~

*tet*(M) *Mho* 2539 ~~~~~~~~~~ ~~~~~~~~~~ ~~~~~~A~~C ~~~~AG~~~~ ~~~~~~~~~~

....|....| ....|....| ....|....| ....|....| ....|....|

955 965 975 985 995

*tet*(M) pMT85-Tet AATTGTTATT TTGCAAAATG AGTTTTTGAA GTTAAATAGT GTTCTTGGAG

*tet*(M) *Mho* 5860 ~~~~~~~~~~ ~~~~~~~~~~ ~~~~~~~~~~ ~~~~~~~~~~ ~~~~~~~~~~

*tet*(M) *Mho* 6892 ~~~~~~~~~~ ~~~~~~~~~~ ~~~~~~~~~~ ~~~~~~~~~~ ~~~~~~~~~~

*tet*(M) *Mho* 6227 ~~~~~~~~~~ ~~~~~~~~~~ ~~~~~~~~~~ ~~~~~~~~~~ ~~~~~~~~~~

*tet*(M) *Mho* 5571 ~~~~~~~~~~ ~~~~~~~~~~ ~~~~~~~~~~ ~~~~~~~~~~ ~~~~~~~~~~

*tet*(M) *Mho* 6585 ~~~~~~~~~~ ~~~~~~~~~~ ~~~~~~~~~~ ~~~~~~~~~~ ~~~~~~~~~~

*tet*(M) *Mho* Sprott ~~~~~~~~~~ ~~~~~~~~~~ ~~~~~~~~~~ ~~~~~~~~~~ ~~~~~~~~~~

*tet*(M) *Mho* 2539 ~~~~~~~~~~ ~~~~~~~~~~ ~~~~~~~~~~ ~~~~~~~~~~ ~~~~~~~~~~

....|....| ....|....| ....|....| ....|....| ....|....|

1005 1015 1025 1035 1045

*tet*(M) pMT85-Tet ATACAAAACT ATTGCCACAG AGAAAAAAGA TTGAAAATCC GCACCCTCTA

*tet*(M) *Mho* 5860 ~~~~~~~~~~ ~~~~~~~~~~ ~~~~~~~~~~ ~~~~~~~~~~ ~~~~~~~~~~

*tet*(M) *Mho* 6892 ~~~~~~~~~~ ~~~~~~~~~~ ~~~~~~~~~~ ~~~~~~~~~~ ~~~~~~~~~~

*tet*(M) *Mho* 6227 ~~~~~~~~~~ ~~~~~~~~~~ ~~~~~~~~~~ ~~~~~~~~~~ ~~~~~~~~~~

*tet*(M) *Mho* 5571 ~~~~~~~~~~ ~~~~~~~~~~ ~~~~~~~~~~ ~~A~~~~~~~ ~~~~~~~~~~

*tet*(M) *Mho* 6585 ~~~~~~~~~~ ~~~~~~~~~~ ~~~~~~~~~~ ~~~~~~~~~~ ~~~~~~~~~~

*tet*(M) *Mho* Sprott ~~~~~~~~~~ ~~~~~~~~~~ ~~~~~~~~~~ ~~~~~~~~~~ ~~~~~~~~~~

*tet*(M) *Mho* 2539 ~~~~~~~~~~ ~~~~~~~~~~ ~~~~~~~~~~ ~~~~~~~~~~ ~~~~~~~~~~

....|....| ....|....| ....|....| ....|....| ....|....|

1055 1065 1075 1085 1095

*tet*(M) pMT85-Tet CTACAAACAA CTGTTGAACC GAGTAAACCT GAACAGAGAG AAATGTTGCT

*tet*(M) *Mho* 5860 ~~~~~~~~~~ ~~~~~~~~~~ ~~~~~~~~~~ ~~~~~~~~~~ ~~~~~~~~~~

*tet*(M) *Mho* 6892 ~~~~~~~~~~ ~~~~~~~~~~ ~~~~~~~~~~ ~~~~~~~~~~ ~~~~~~~~~~

*tet*(M) *Mho* 6227 ~~~~~~~~~~ ~~~~~~~~~~ ~~~~~~~~~~ ~~~~~~~~~~ ~~~~~~~~~~

*tet*(M) *Mho* 5571 ~~~~~~~~~~ ~~~~~~~~~~ ~~~~~~~~~~ ~~~~~~~~~~ ~~~~~~~~~~

*tet*(M) *Mho* 6585 ~~~~~~~~~~ ~~~~~~~~~~ ~~~~~~~~~~ ~~~~~~~~~~ ~~~~~~~~~~

*tet*(M) *Mho* Sprott ~~~~~~~~~~ ~~~~~~~~~~ ~~~~~~~~~~ ~~~~~~~~~~ ~~~~~~~~~~

*tet*(M) *Mho* 2539 ~~~~~~~~~~ ~~~~~~~~~~ ~~~~~~~~~~ ~~~~~~~~~~ ~~~~~~~~~~

....|....| ....|....| ....|....| ....|....| ....|....|

1105 1115 1125 1135 1145

*tet*(M) pMT85-Tet TGATGCCCTT TTGGAAATCT CAGATAGTGA TCCGCTTCTA CGATATTACG

*tet*(M) *Mho* 5860 ~~~~~~~~~~ ~~~~~~~~~~ ~~~~~~~~~~ ~~~~~~~~~~ ~~~~~~~~~~

*tet*(M) *Mho* 6892 ~~~~~~~~~~ ~~~~~~~~~~ ~~~~~~~~~~ ~~~~~~~~~~ ~~~~~~~~~~

*tet*(M) *Mho* 6227 ~~~~~~~~~~ ~~~~~~~~~~ ~~~~~~~~~~ ~~~~~~~~~~ ~~~~~~~~~~

*tet*(M) *Mho* 5571 ~~~~~~~~~~ ~~~~~~~~~~ ~~~~~~~~~~ ~~~~~~~~~~ ~~~~~~~~~~

*tet*(M) *Mho* 6585 ~~~~~~~~~~ ~~~~~~~~~~ ~~~~~~~~~~ ~~~~~~~~~~ ~~~~~~~~~~

*tet*(M) *Mho* Sprott ~~~~~~~~~~ ~~~~~~~~~~ ~~~~~~~~~~ ~~~~~~~~~~ ~~~~~~~~~~

*tet*(M) *Mho* 2539 ~~~~~~~~~~ ~~~~~~~~~~ ~~~~~~~~~~ ~~~~~~~~~~ ~~~~~~~~~~

....|....| ....|....| ....|....| ....|....| ....|....|

1155 1165 1175 1185 1195

*tet*(M) pMT85-Tet TGGATTCTAC GACACATGAA ATTATACTTT CTTTCTTAGG GAAAGTACAA

*tet*(M) *Mho* 5860 ~~~~~~~~~~ ~~~~~~~~~~ ~~~~~~~~~~ ~~~~~~~~~~ ~~~~~~~~~~

*tet*(M) *Mho* 6892 ~~~~~~~~~~ ~~~~~~~~~~ ~~~~~~~~~~ ~~~~~~~~~~ ~~~~~~~~~~

*tet*(M) *Mho* 6227 ~~~~~~~~~~ ~~~~~~~~~~ ~~~~~~~~~~ ~~~~~~~~~~ ~~~~~~~~~~

*tet*(M) *Mho* 5571 ~~~~~~~~~~ ~~~~~~~~~~ ~~~~~~~~~~ ~~~~~~~~~~ ~~~~~~~~~~

*tet*(M) *Mho* 6585 ~~~~~~~~~~ ~~~~~~~~~~ ~~~~~~~~~~ ~~~~~~~~~~ ~~~~~~~~~~

*tet*(M) *Mho* Sprott ~~~~~~~~~~ ~~~~~~~~~~ ~~~~~~~~~~ ~~~~~~~~~~ ~~~~~~~~~~

*tet*(M) *Mho* 2539 ~~~~~~~~~~ ~~~~~~~~~~ ~~~~~~~~~~ ~~~~~~~~~~ ~~~~~~~~~~

....|....| ....|....| ....|....| ....|....| ....|....|

1205 1215 1225 1235 1245

*tet*(M) pMT85-Tet ATGGAAGTGA TTAGTGCACT GTTGCAAGAA AAGTATCATG TGGAGATAGA

*tet*(M) *Mho* 5860 ~~~~~~~~~~ ~~~~~~~~~~ ~~~~~~~~~~ ~~~~~~~~~~ ~~~~~~~~~~

*tet*(M) *Mho* 6892 ~~~~~~~~~~ ~~~~~~~~~~ ~~~~~~~~~~ ~~~~~~~~~~ ~~~~~~~~~~

*tet*(M) *Mho* 6227 ~~~~~~~~~~ ~~~~~~~~~~ ~~~~~~~~~~ ~~~~~~~~~~ ~~~~~~~~~~

*tet*(M) *Mho* 5571 ~~~~~~~~~~ ~~~~~~~~~~ ~~~~~~~~~~ ~~~~~~~~~~ ~~~~~~~~~~

*tet*(M) *Mho* 6585 ~~~~~~~~~~ ~~~~~~~~~~ ~~~~~~~~~~ ~~~~~~~~~~ ~~~~~~~~~~

*tet*(M) *Mho* Sprott ~~~~~~~~~~ ~~~~~~~~~~ ~~~~~~~~~~ ~~~~~~~~~~ ~~~~~~~~~~

*tet*(M) *Mho* 2539 ~~~~~~~~~~ ~~~~~~~~~~ ~~~~~~~~~~ ~~~~~~~~~~ ~~~~~~~~~~

....|....| ....|....| ....|....| ....|....| ....|....|

1255 1265 1275 1285 1295

*tet*(M) pMT85-Tet ACTAAAAGAG CCTACAGTCA TTTATATGGA GAGACCGTTA AAAAATGCAG

*tet*(M) *Mho* 5860 ~~~~~~~~~~ ~~~~~~~~~~ ~~~~~~~~~~ ~~~~~~~~~~ ~~~~~~~~~~

*tet*(M) *Mho* 6892 ~~~~~~~~~~ ~~~~~~~~~~ ~~~~~~~~~~ ~~~~~~~~~~ ~~~~~~~~~~

*tet*(M) *Mho* 6227 ~~~~~~~~~~ ~~~~~~~~~~ ~~~~~~~~~~ ~~~~~~~~~~ ~~~~~~~~~~

*tet*(M) *Mho* 5571 ~~~~~~~~~~ ~~~~~~~~~~ ~~~~~~~~~~ ~~~~~~~~~~ ~~~~~~~~~~

*tet*(M) *Mho* 6585 ~~~~~~~~~~ ~~~~~~~~~~ ~~~~~~~~~~ ~~~~~~~~~~ ~~~~~~~~~~

*tet*(M) *Mho* Sprott ~~~~~~~~~~ ~~~~~~~~~~ ~~~~~~~~~~ ~~~~~~~~~~ ~~~~~~~~~~

*tet*(M) *Mho* 2539 ~~~~~~~~~~ ~~~~~~~~~~ ~~~~~~~~~~ ~~~~~~~~~~ ~~~~~~~~~~

....|....| ....|....| ....|....| ....|....| ....|....|

1305 1315 1325 1335 1345

*tet*(M) pMT85-Tet AATATACCAT TCACATCGAA GTGCCGCCAA ATCCTTTCTG GGCTTCCATT

*tet*(M) *Mho* 5860 ~~~~~~~~~~ ~~~~~~~~~~ ~~~~~~~~~~ ~~~~~~~~~~ ~~~~~~~~~~

*tet*(M) *Mho* 6892 ~~~~~~~~~~ ~~~~~~~~~~ ~~~~~~~~~~ ~~~~~~~~~~ ~~~~~~~~~~

*tet*(M) *Mho* 6227 ~~~~~~~~~~ ~~~~~~~~~~ ~~~~~~~~~~ ~~~~~~~~~~ ~~~~~~~~~~

*tet*(M) *Mho* 5571 ~~~~~~~~~~ ~~~~~~~~~~ ~~~~~~~~~~ ~~~~~~~~~~ ~~~~~~~~~~

*tet*(M) *Mho* 6585 ~~~~~~~~~~ ~~~~~~~~~~ ~~~~~~~~~~ ~~~~~~~~~~ ~~~~~~~~~~

*tet*(M) *Mho* Sprott ~~~~~~~~~~ ~~~~~~~~~~ ~~~~~~~~~~ ~~~~~~~~~~ ~~~~~~~~~~

*tet*(M) *Mho* 2539 ~~~~~~~~~~ ~~~~~~~~~~ ~~~~~~~~~~ ~~~~~~~~~~ ~~~~~~~~~~

....|....| ....|....| ....|....| ....|....| ....|....|

1355 1365 1375 1385 1395

*tet*(M) pMT85-Tet GGTTTATCTG TATCACCGCT TCCGTTGGGA AGTGGAATGC AGTATGAGAG

*tet*(M) *Mho* 5860 ~~~~~~~~~~ ~~~~~~~~~~ ~~~~~~~~~~ ~~~~~~~~~~ ~~~~~~~~~~

*tet*(M) *Mho* 6892 ~~~~~~~~~~ ~~~~~~~~~~ ~~~~~~~~~~ ~~~~~~~~~~ ~~~~~~~~~~

*tet*(M) *Mho* 6227 ~~~~~~~~~~ ~~~~~~~~~~ ~~~~~~~~~~ ~~~~~~~~~~ ~~~~~~~~~~

*tet*(M) *Mho* 5571 ~~~~~~~~~~ ~~~~~~~~~~ ~~~~~~~~~~ ~~~~~~~~~~ ~~~~~~~~~~

*tet*(M) *Mho* 6585 ~~~~~~~~~~ ~~~~~~~~~~ ~~~~~~~~~~ ~~~~~~~~~~ ~~~~~~~~~~

*tet*(M) *Mho* Sprott ~~~~~~~~~~ ~~~~~~~~~~ ~~~~~~~~~~ ~~~~~~~~~~ ~~~~~~~~~~

*tet*(M) *Mho* 2539 ~~~~~~~~~~ ~~~~~~~~~~ ~~~~~~~~~~ ~~~~~~~~~~ ~~~~~~~~~~

....|....| ....|....| ....|....| ....|....| ....|....|

1405 1415 1425 1435 1445

*tet*(M) pMT85-Tet CTCGGTTTCT CTTGGATACT TAAATCAATC ATTTCAAAAT GCAGTTATGG

*tet*(M) *Mho* 5860 ~~~~~~~~~~ ~~~~~~~~~~ ~~~~~~~~~~ ~~~~~~~~~~ ~~~~~~~~~~

*tet*(M) *Mho* 6892 ~~~~~~~~~~ ~~~~~~~~~~ ~~~~~~~~~~ ~~~~~~~~~~ ~~~~~~~~~~

*tet*(M) *Mho* 6227 ~~~~~~~~~~ ~~~~~~~~~~ ~~~~~~~~~~ ~~~~~~~~~~ ~~~~~~~~~~

*tet*(M) *Mho* 5571 ~~~~~~~~~~ ~~~~~~~~~~ ~~~~~~~~~~ ~~~~~~~~~~ ~~~~~~~~~~

*tet*(M) *Mho* 6585 ~~~~~~~~~~ ~~~~~~~~~~ ~~~~~~~~~~ ~~~~~~~~~~ ~~~~~~~~~~

*tet*(M) *Mho* Sprott ~~~~~~~~~~ ~~~~~~~~~~ ~~~~~~~~~~ ~~~~~~~~~~ ~~~~~~~~~~

*tet*(M) *Mho* 2539 ~~~~~~~~~~ ~~~~~~~~~~ ~~~~~~~~~~ ~~~~~~~~~~ ~~~~~~~~~~

....|....| ....|....| ....|....| ....|....| ....|....|

1455 1465 1475 1485 1495

*tet*(M) pMT85-Tet AAGGGATACG CTATGGTTGC GAACAAGGAT TATATGGTTG GAATGTGACG

*tet*(M) *Mho* 5860 ~~~~~~~~~~ ~~~~~~~~~~ ~~~~~~~~~~ ~~~~~~~~~~ ~~~~~~~~~~

*tet*(M) *Mho* 6892 ~~~~~~~~~~ ~~~~~~~~~~ ~~~~~~~~~~ ~~~~~~~~~~ ~~~~~~~~~~

*tet*(M) *Mho* 6227 ~~~~~~~~~~ ~~~~~~~~~~ ~~~~~~~~~~ ~~~~~~~~~~ ~~~~~~~~~~

*tet*(M) *Mho* 5571 ~~~~~~~~~~ ~~~~~~~~~~ ~~~~~~~~~~ ~~~~~~~~~~ ~~~~~~~~~~

*tet*(M) *Mho* 6585 ~~~~~~~~~~ ~~~~~~~~~~ ~~~~~~~~~~ ~~~~~~~~~~ ~~~~~~~~~~

*tet*(M) *Mho* Sprott ~~~~~~~~~~ ~~~~~~~~~~ ~~~~~~~~~~ ~~~~~~~~~~ ~~~~~~~~~~

*tet*(M) *Mho* 2539 ~~~~~~~~~~ ~~~~~~~~~~ ~~~~~~~~~~ ~~~~~~~~~~ ~~~~~~~~~~

....|....| ....|....| ....|....| ....|....| ....|....|

1505 1515 1525 1535 1545

*tet*(M) pMT85-Tet GATTGTAAAA TCTGTTTTAA GTATGGCTTA TACTATAGCC CTGTTAGTAC

*tet*(M) *Mho* 5860 ~~C~~~~~~~ ~~~~~~~~~~ ~~~~~~~~~~ ~~~~~~~~~~ ~~~~~~~~~~

*tet*(M) *Mho* 6892 ~~C~~~~~~~ ~~~~~~~~~~ ~~~~~~~~~~ ~~~~~~~~~~ ~~~~~~~~~~

*tet*(M) *Mho* 6227 ~~C~~~~~~~ ~~~~~~~~~~ ~~~~~~~~~~ ~~~~~~~~~~ ~~~~~~~~~~

*tet*(M) *Mho* 5571 ~~C~~~~~~~ ~~~~~~~~~~ ~~~~~~~~~~ ~~~~~~~~~~ ~~~~~~~~~~

*tet*(M) *Mho* 6585 ~~C~~~~~~~ ~~~~~~~~~~ ~~~~~~~~~~ ~~~~~~~~~~ ~~~~~~~~~~

*tet*(M) *Mho* Sprott ~~C~~~~~~~ ~~~~~~~~~~ ~~~~~~~~~~ ~~~~~~~~~~ ~~~~~~~~~~

*tet*(M) *Mho* 2539 ~~C~~~~~~~ ~~~~~~~~~~ ~~~~~~~~~~ ~~~~~~~~~~ ~~~~~~~~~~

....|....| ....|....| ....|....| ....|....| ....|....|

1555 1565 1575 1585 1595

*tet*(M) pMT85-Tet CCCAGCAGAT TTTCGGATGC TTGCTCCTAT TGTATTGGAA CAAGTCTTAA

*tet*(M) *Mho* 5860 ~~~~~~~~~~ ~~~~~~~~~~ ~~~~~~~~~~ ~~~~~~~~~~ ~~~~~~~~~~

*tet*(M) *Mho* 6892 ~~~~~~~~~~ ~~~~~~~~~~ ~~~~~~~~~~ ~~~~~~~~~~ ~~~~~~~~~~

*tet*(M) *Mho* 6227 ~~~~~~~~~~ ~~~~~~~~~~ ~~~~~~~~~~ ~~~~~~~~~~ ~~~~~~~~~~

*tet*(M) *Mho* 5571 ~~~~~~~~~~ ~~~~~~~~~~ ~~~~~~~~~~ ~~~~~~~~~~ ~~~~~~~~~~

*tet*(M) *Mho* 6585 ~~~~~~~~~~ ~~~~~~~~~~ ~~~~~~~~~~ ~~~~~~~~~~ ~~~~~~~~~~

*tet*(M) *Mho* Sprott ~~~~~~~~~~ ~~~~~~~~~~ ~~~~~~~~~~ ~~~~~~~~~~ ~~~~~~~~~~

*tet*(M) *Mho* 2539 ~~~~~~~~~~ ~~~~~~~~~~ ~~~~~~~~~~ ~~~~~~~~~~ ~~~~~~~~~~

....|....| ....|....| ....|....| ....|....| ....|....|

1605 1615 1625 1635 1645

*tet*(M) pMT85-Tet AAAAAGCTGG AACAGAATTG TTAGAGCCAT ATCTTAGTTT TAAAATTTAT

*tet*(M) *Mho* 5860 ~~~~~~~~~~ ~~~~~~~~~~ ~~~~~~~~~~ ~~~~~~~~~~ ~~~~~~~~~~

*tet*(M) *Mho* 6892 ~~~~~~~~~~ ~~~~~~~~~~ ~~~~~~~~~~ ~~~~~~~~~~ ~~~~~~~~~~

*tet*(M) *Mho* 6227 ~~~~~~~~~~ ~~~~~~~~~~ ~~~~~~~~~~ ~~~~~~~~~~ ~~~~~~~~~~

*tet*(M) *Mho* 5571 ~~~~~~~~~~ ~~~~~~~~~~ ~~~~~~~~~~ ~~~~~~~~~~ ~~~~~~~~~~

*tet*(M) *Mho* 6585 ~~~~~~~~~~ ~~~~~~~~~~ ~~~~~~~~~~ ~~~~~~~~~~ ~~~~~~~~~~

*tet*(M) *Mho* Sprott ~~~~~~~~~~ ~~~~~~~~~~ ~~~~~~~~~~ ~~~~~~~~~~ ~~~~~~~~~~

*tet*(M) *Mho* 2539 ~~~~~~~~~~ ~~~~~~~~~~ ~~~~~~~~~~ ~~~~~~~~~~ ~~~~~~~~~~

....|....| ....|....| ....|....| ....|....| ....|....|

1655 1665 1675 1685 1695

*tet*(M) pMT85-Tet GCGCCACAGG AATATCTTTC ACGAGCATAC AACGATGCTC CTAAATATTG

*tet*(M) *Mho* 5860 ~~~~~~~~~~ ~~~~~~~~~T ~~~~~~~~~~ ~~~~~~~~~~ ~~~~~~~~~~

*tet*(M) *Mho* 6892 ~~~~~~~~~~ ~~~~~~~~~T ~~~~~~~~~~ ~~~~~~~~~~ ~~~~~~~~~~

*tet*(M) *Mho* 6227 ~~~~~~~~~~ ~~~~~~~~~~ ~~~~~~~~~~ ~~~~~~~~~~ ~~~~~~~~~~

*tet*(M) *Mho* 5571 ~~~~~~~~~~ ~~~~~~~~~~ ~~~~~~~~~~ ~~~~~~~~~~ ~~~~~~~~~~

*tet*(M) *Mho* 6585 ~~~~~~~~~~ ~~~~~~~~~~ ~~~~~~~~~~ ~~~~~~~~~~ ~~~~~~~~~~

*tet*(M) *Mho* Sprott ~~~~~~~~~~ ~~~~~~~~~~ ~~~~~~~~~~ ~~~~~~~~~~ ~~~~~~~~~~

*tet*(M) *Mho* 2539 ~~~~~~~~~~ ~~~~~~~~~~ ~~~~~~~~~~ ~~~~~~~~~~ ~~~~~~~~~~

....|....| ....|....| ....|....| ....|....| ....|....|

1705 1715 1725 1735 1745

*tet*(M) pMT85-Tet TGCGAACATC GTAGACACTC AATTGAAAAA TAATGAGGTC ATTCTTAGTG

*tet*(M) *Mho* 5860 ~~~~~~~~~~ ~~~~~~~~~~ ~~~~~~~~~~ ~~~~~~~~~~ ~~~~~~~~~~

*tet*(M) *Mho* 6892 ~~~~~~~~~~ ~~~~~~~~~~ ~~~~~~~~~~ ~~~~~~~~~~ ~~~~~~~~~~

*tet*(M) *Mho* 6227 ~~~~~~~~~~ ~~~~~~~~~~ ~~~~~~~~~~ ~~~~~~~~~~ ~~~~~~~~~~

*tet*(M) *Mho* 5571 ~~~~~~~~~~ ~~~~~~~~~~ ~~~~~~~~~~ ~~~~~~~~~~ ~~~~~~~~~~

*tet*(M) *Mho* 6585 ~~~~~~~~~~ ~~~~~~~~~~ ~~~~~~~~~~ ~~~~~~~~~~ ~~~~~~~~~~

*tet*(M) *Mho* Sprott ~~~~~~~~~~ ~~~~~~~~~~ ~~~~~~~~~~ ~~~~~~~~~~ ~~~~~~~~~~

*tet*(M) *Mho* 2539 ~~~~~~~~~~ ~~~~~~~~~~ ~~~~~~~~~~ ~~~~~~~~~~ ~~~~~~~~~~

....|....| ....|....| ....|....| ....|....| ....|....|

1755 1765 1775 1785 1795

*tet*(M) pMT85-Tet GAGAAATCCC TGCTCGGTGT ATTCAAGAAT ATCGTAGTGA TTTAACTTTC

*tet*(M) *Mho* 5860 ~~~~~~~~~~ ~~~~~~~~~~ ~~~~~~~~~~ ~~~~~~~~~~ ~~~~~~~~~~

*tet*(M) *Mho* 6892 ~~~~~~~~~~ ~~~~~~~~~~ ~~~~~~~~~~ ~~~~~~~~~~ ~~~~~~~~~~

*tet*(M) *Mho* 6227 ~~~~~~~~~~ ~~~~~~~~~~ ~~~~~~~~~~ ~~~~~~~~~~ ~~~~~~~~~~

*tet*(M) *Mho* 5571 ~~~~~~~~~~ ~~~~~~~~~~ ~~~~~~~~~~ ~~~~~~~~~~ ~~~~~~~~~~

*tet*(M) *Mho* 6585 ~~~~~~~~~~ ~~~~~~~~~~ ~~~~~~~~~~ ~~~~~~~~~~ ~~~~~~~~~~

*tet*(M) *Mho* Sprott ~~~~~~~~~~ ~~~~~~~~~~ ~~~~~~~~~~ ~~~~~~~~~~ ~~~~~~~~~~

*tet*(M) *Mho* 2539 ~~~~~~~~~~ ~~~~~~~~~~ ~~~~~~~~~~ ~~~~~~~~~~ ~~~~~~~~~~

....|....| ....|....| ....|....| ....|....| ....|....|

1805 1815 1825 1835 1845

*tet*(M) pMT85-Tet TTTACAAATG GACGTAGTGT TTGTTTAACA GAGTTAAAAG GGTACCATGT

*tet*(M) *Mho* 5860 ~~~~~~~~~~ ~~~~~~~~~~ ~~~~~~~~~~ ~~~~~~~~~~ ~~~~~~~~~~

*tet*(M) *Mho* 6892 ~~~~~~~~~~ ~~~~~~~~~~ ~~~~~~~~~~ ~~~~~~~~~~ ~~~~~~~~~~

*tet*(M) *Mho* 6227 ~~~~~~~~~~ ~~~~~~~~~~ ~~~~~~~~~~ ~~~~~~~~~~ ~~~~~~~~~~

*tet*(M) *Mho* 5571 ~~~~~~~~~~ ~~~~~~~~~~ ~~~~~~~~~~ ~~~~~~~~~~ ~~~~~~~~~~

*tet*(M) *Mho* 6585 ~~~~~~~~~~ ~~~~~~~~~~ ~~~~~~~~~~ ~~~~~~~~~~ ~~~~~~~~~~

*tet*(M) *Mho* Sprott ~~~~~~~~~~ ~~~~~~~~~~ ~~~~~~~~~~ ~~~~~~~~~~ ~~~~~~~~~~

*tet*(M) *Mho* 2539 ~~~~~~~~~~ ~~~~~~~~~~ ~~~~~~~~~~ ~~~~~~~~~~ ~~~~~~~~~~

....|....| ....|....| ....|....| ....|....| ....|....|

1855 1865 1875 1885 1895

*tet*(M) pMT85-Tet TACTACCGGT GAACCTGTTT GCCAGCCCCG TCGTCCAAAT AGTCGGATAG

*tet*(M) *Mho* 5860 ~~~~~~~~~~ ~~~~~~~~~~ ~~~~~~~~~~ ~~~~~~~~~~ ~~~~~~~~~~

*tet*(M) *Mho* 6892 ~~~~~~~~~~ ~~~~~~~~~~ ~~~~~~~~~~ ~~~~~~~~~~ ~~~~~~~~~~

*tet*(M) *Mho* 6227 ~~~~~~~~~~ ~~~~~~~~~~ ~~~~~~~~~~ ~~~~~~~~~~ ~~~~~~~~~~

*tet*(M) *Mho* 5571 ~~~~~~~~~~ ~~~~~~~~~~ ~~~~~~~~~~ ~~~~~~~~~~ ~~~~~~~~~~

*tet*(M) *Mho* 6585 ~~~~~~~~~~ ~~~~~~~~~~ ~~~~~~~~~~ ~~~~~~~~~~ ~~~~~~~~~~

*tet*(M) *Mho* Sprott ~~~~~~~~~~ ~~~~~~~~~~ ~~~~~~~~~~ ~~~~~~~~~~ ~~~~~~~~~~

*tet*(M) *Mho* 2539 ~~~~~~~~~~ ~~~~~~~~~~ ~~~~~~~~~~ ~~~~~~~~~~ ~~~~~~~~~~

....|....| ....|....| ....|....| ....|

1905 1915 1925 1935

*tet*(M) pMT85-Tet ATAAAGTACG ATATATGTTC AATAAAATAA CTTAG

*tet*(M) *Mho* 5860 ~~~~~~~~~~ ~~~~~~~~~~ ~~~~~~~~~~ ~~~~~

*tet*(M) *Mho* 6892 ~~~~~~~~~~ ~~~~~~~~~~ ~~~~~~~~~~ ~~~~~

*tet*(M) *Mho* 6227 ~~~~~~~~~~ ~~~~~~~~~~ ~~~~~~~~~~ ~~~~~

*tet*(M) *Mho* 5571 ~~~~~~~~~~ ~~~~~~~~~~ ~~~~~~~~~~ ~~~~~

*tet*(M) *Mho* 6585 ~~~~~~~~~~ ~~~~~~~~~~ ~~~~~~~~~~ ~~~~~

*tet*(M) *Mho* Sprott ~~~~~~~~~~ ~~~~~~~~~~ ~~~~~~~~~~ ~~~~~

*tet*(M) *Mho* 2539 ~~~~~~~~~~ ~~~~~~~~~~ ~~~~~~~~~~ ~~~~~

**b.** Alignment of Tet(M) amino acid sequences of the pMT85-Tet plasmid and seven *M*.*hominis* (*Mho*) clinical isolates resistant to tetracycline (5860, 6892, 6227, 5571, 6585 (Meygret *et al*, 2018, J Antimicrob Chemother (22)), Sprott [accession number CP011538.1], and 2539 [accession number NZ_CP026341.1]). The consensus amino acid sequence corresponding to that of the pMT85-Tet plasmid (*Enterococcus*-derived) is indicated above and indication of amino acid changes is provided below (n=13).

....|....| ....|....| ....|....| ....|....| ....|....|

10 20 30 40 50

TetM pMT85-Tet .....MKIIN IGVLAHVDAG KTTLTESLLY NSGAITELGS VDKGTTRTDN

TetM *Mho* 5860 .....~~~~~ ~~~~~~~~~~ ~~~~~~~~~~ ~~~~~~~~~~ ~~~~~~~~~~

TetM *Mho* 6892 .....~~~~~ ~~~~~~~~~~ ~~~~~~~~~~ ~~~~~~~~~~ ~~~~~~~~~~

TetM *Mho* 5571 .....~~~~~ ~~~~~~~~~~ ~~~~~~~~~~ ~~~~~~~~~~ ~~~~~~~~~~

TetM *Mho* 6227 .....~~~~~ ~~~~~~~~~~ ~~~~~~~~~~ ~~~~~~~~~~ ~~~~~~~~~~

TetM *Mho* 6585 .....~~~~~ ~~~~~~~~~~ ~~~~~~~~~~ ~~~~~~~~~~ ~~~~~~~~~~

TetM *Mho* Sprott .....~~~~~ ~~~~~~~~~~ ~~~~~~~~~~ ~~~~~~~~~~ ~~~~~~~~~~

TetM *Mho* 2539 .....~~~~~ ~~~~~~~~~~ ~~~~~~~~~~ ~~~~~~~~~~ ~~~~~~~~~~

....|....| ....|....| ....|....| ....|....| ....|....|

60 70 80 90 100

TetM pMT85-Tet TLLERQRGIT IQTGITSFQW ENTKVNIIDT PGHMDFLAEV YRSLSVLDGA

TetM *Mho* 5860 ~~~~~~~~~~ ~~~~~~~~~~ ~~~~~~~~~~ ~~~~~~~~~~ ~~~~~~~~~~

TetM *Mho* 6892 ~~~~~~~~~~ ~~~~~~~~~~ ~~~~~~~~~~ ~~~~~~~~~~ ~~~~~~~~~~

TetM *Mho* 5571 ~~~~~~~~~~ ~~~~~~~~~~ ~~~~~~~~~~ ~~~~~~~~~~ ~~~~~~~~~~

TetM *Mho* 6227 ~~~~~~~~~~ ~~~~~~~~~~ ~~~~~~~~~~ ~~~~~~~~~~ ~~~~~~~~~~

TetM *Mho* 6585 ~~~~~~~~~~ ~~~~~~~~~~ ~~~~~~~~~~ ~~~~~~~~~~ ~~~~~~~~~~

TetM *Mho* Sprott ~~~~~~~~~~ ~~~~~~~~~~ ~~~~~~~~~~ ~~~~~~~~~~ ~~~~~~~~~~

TetM *Mho* 2539 ~~~~~~~~~~ ~~~~~~~~~~ ~~~~~~~~~~ ~~~~~~~~~~ ~~~~~~~~~~

....|....| ....|....| ....|....| ....|....| ....|....|

110 120 130 140 150

TetM pMT85-Tet ILLISAKDGV QAQTRILFHA LRKMGIPTIF FINKIDQNGI DLSTVYQDIK

TetM *Mho* 5860 ~~~~~~~~~~ ~~~~~~~~~~ ~~~~~~~~~~ ~~~~~~~~~~ ~~~~~~~~~~

TetM *Mho* 6892 ~~~~~~~~~~ ~~~~~~~~~~ ~~~~~~~~~~ ~~~~~~~~~~ ~~~~~~~~~~

TetM *Mho* 5571 ~~~~~~~~~~ ~~~~~~~~~~ ~~~~~~~~~~ ~~~~~~~~~~ ~~~~~~~~~~

TetM *Mho* 6227 ~~~~~~~~~~ ~~~~~~~~~~ ~~~~~~~~~~ ~~~~~~~~~~ ~~~~~~~~~~

TetM *Mho* 6585 ~~~~~~~~~~ ~~~~~~~~~~ ~~~~~~~~~~ ~~~~~~~~~~ ~~~~~~~~~~

TetM *Mho* Sprott ~~~~~~~~~~ ~~~~~~~~~~ ~~~~~~~~~~ ~~~~~~~~~~ ~~~~~~~~~~

TetM *Mho* 2539 ~~~~~~~~~~ ~~~~~~~~~~ ~~~~~~~~~~ ~~~~~~~~~~ ~~~~~~~~~~

....|....| ....|....| ....|....| ....|....| ....|....|

160 170 180 190 200

TetM pMT85-Tet EKLSAEIVIK QKVELYPNMC VTNFTESEQW DTVIEGNDDL LEKYMSGKSL

TetM *Mho* 5860 ~~~~~~~~~~ ~~~~~~~~~~ ~~~~~~~~~~ ~~~~~~~~~~ ~~~~L~~~~~

TetM *Mho* 6892 ~~~~~~~~~~ ~~~~~~~~~~ ~~~~~~~~~~ ~~~~~~~~~~ ~~~~~~~~~~

TetM *Mho* 5571 ~~~~~~~~~~ ~~~~~~~~~~ ~~~~~~~~~~ ~~~~~~~~~~ ~~~~~~~~~~

TetM *Mho* 6227 ~~~~~~~~~~ ~~~~~~~~~~ ~~~~~~~~~~ ~~~~~~~~~~ ~~~~~~~~~~

TetM *Mho* 6585 ~~~~~~~~~~ ~~~~~~~~~~ ~~~~~~~~~~ ~~~~~~~~~~ ~~~~~~~~~~

TetM *Mho* Sprott ~~~~~~~~~~ ~~~~~~~~~~ ~~~~~~~~~~ ~~~~~~~~~~ ~~~~~~~~~~

TetM *Mho* 2539 ~~~~~~~~~~ ~~~~~~~~~~ ~~~~~~~~~~ ~~~~~~~~~~ ~~~~~~~~~~

....|....| ....|....| ....|....| ....|....| ....|....|

210 220 230 240 250

TetM pMT85-Tet EALELEQEES IRFHNCSLFP VYHGSAKNNI GIDNLIEVIT NKFYSSTHRG

TetM *Mho* 5860 ~~~~~~~~~~ ~~~Q~~~~~~ ~~~~~~~~~~ ~~~~~~~~~~ ~~~~~~~~~~

TetM *Mho* 6892 ~~~~~~~~~~ ~~~Q~~~~~~ ~~~~~~~~~~ ~~~~~~~~~~ ~~~~~~~~~~

TetM *Mho* 5571 ~~~~~~~~~~ ~~~Q~~~~~~ ~~~~~~~~~~ ~~~~~~~~~~ ~~~~~~~~~~

TetM *Mho* 6227 ~~~~~~~~~~ ~~~Q~~~~~~ ~~~~~~~~~~ ~~~~~~~~~~ ~~~~~~~~~~

TetM *Mho* 6585 ~~~~~~~~~~ ~~~Q~~~~~~ ~~~~~~~~~~ ~~~~~~~~~~ ~~~~~~~~~~

TetM *Mho* Sprott ~~~~~~~~~~ ~~~Q~~~~~~ ~~~~~~~~~~ ~~~~~~~~~~ ~~~~~~~~~~

TetM *Mho* 2539 ~~~~~~~~~~ ~~~Q~~~~~~ ~~~~~~~~~~ ~~~~~~~~~~ ~~~~~~~~~~

....|....| ....|....| ....|....| ....|....| ....|....|

260 270 280 290 300

TetM pMT85-Tet PSELCGNVFK IEYTKKRQRL AYIRLYSGVL HLRDSVRVSE KEKIKVTEMY

TetM *Mho* 5860 Q~~~~~K~~~ ~~~SE~~~~~ ~~~~~~~~~~ ~~~~~~~I~~ ~~~~~I~~~~

TetM *Mho* 6892 Q~~~~~K~~~ ~~~SE~~~~~ ~~~~~~~~~~ ~~~~~~~I~~ ~~~~~I~~~~

TetM *Mho* 5571 Q~~~~~K~~~ ~~~SE~~~~~ ~~~~~~~~~~ ~~~~~~~I~~ ~~~~~II~~~

TetM *Mho* 6227 Q~~~~~K~~~ ~~~SE~~~~~ ~~~~~~~~~~ ~~~~L~~I~~ ~~~~~I~~~~

TetM *Mho* 6585 Q~~~~~K~~~ ~~~SE~~~~~ ~~~~~~~~~~ ~~~~~~~I~~ ~~~~~I~~~~

TetM *Mho* Sprott Q~~~~~K~~~ ~~~SE~~~~~ ~~~~~~~~~~ ~~~~L~~I~~ ~~~~~I~~~~

TetM *Mho* 2539 Q~~~~~K~~~ ~~~SE~~~~~ ~~~~~~~~~~ ~~~~L~~I~~ ~~~~~I~~~~

....|....| ....|....| ....|....| ....|....| ....|....|

310 320 330 340 350

TetM pMT85-Tet TSINGELCKI DRAYSGEIVI LQNEFLKLNS VLGDTKLLPQ RKKIENPHPL

TetM *Mho* 5860 ~~~~~~~~~~ ~K~~~~~~~~ ~~~~~~~~~~ ~~~~~~~~~~ ~~~~~~~~~~

TetM *Mho* 6892 ~~~~~~~~~~ ~K~~~~~~~~ ~~~~~~~~~~ ~~~~~~~~~~ ~~~~~~~~~~

TetM *Mho* 5571 ~~~~~~~~~~ ~K~~~~~~~~ ~~~~~~~~~~ ~~~~~~~~~~ ~~~~K~~~~~

TetM *Mho* 6227 ~~~~~~~~~~ ~K~~~~~~~~ ~~~~~~~~~~ ~~~~~~~~~~ ~~~~~~~~~~

TetM *Mho* 6585 ~~~~~~~~~~ ~K~~~~~~~~ ~~~~~~~~~~ ~~~~~~~~~~ ~~~~~~~~~~

TetM *Mho* Sprott ~~~~~~~~~~ ~K~~~~~~~~ ~~~~~~~~~~ ~~~~~~~~~~ ~~~~~~~~~~

TetM *Mho* 2539 ~~~~~~~~~~ ~K~~~~~~~~ ~~~~~~~~~~ ~~~~~~~~~~ ~~~~~~~~~~

....|....| ....|....| ....|....| ....|....| ....|....|

360 370 380 390 400

TetM pMT85-Tet LQTTVEPSKP EQREMLLDAL LEISDSDPLL RYYVDSTTHE IILSFLGKVQ

TetM *Mho* 5860 ~~~~~~~~~~ ~~~~~~~~~~ ~~~~~~~~~~ ~~~~~~~~~~ ~~~~~~~~~~

TetM *Mho* 6892 ~~~~~~~~~~ ~~~~~~~~~~ ~~~~~~~~~~ ~~~~~~~~~~ ~~~~~~~~~~

TetM *Mho* 5571 ~~~~~~~~~~ ~~~~~~~~~~ ~~~~~~~~~~ ~~~~~~~~~~ ~~~~~~~~~~

TetM *Mho* 6227 ~~~~~~~~~~ ~~~~~~~~~~ ~~~~~~~~~~ ~~~~~~~~~~ ~~~~~~~~~~

TetM *Mho* 6585 ~~~~~~~~~~ ~~~~~~~~~~ ~~~~~~~~~~ ~~~~~~~~~~ ~~~~~~~~~~

TetM *Mho* Sprott ~~~~~~~~~~ ~~~~~~~~~~ ~~~~~~~~~~ ~~~~~~~~~~ ~~~~~~~~~~

TetM *Mho* 2539 ~~~~~~~~~~ ~~~~~~~~~~ ~~~~~~~~~~ ~~~~~~~~~~ ~~~~~~~~~~

....|....| ....|....| ....|....| ....|....| ....|....|

410 420 430 440 450

TetM pMT85-Tet MEVISALLQE KYHVEIELKE PTVIYMERPL KNAEYTIHIE VPPNPFWASI

TetM *Mho* 5860 ~~~~~~~~~~ ~~~~~~~~~~ ~~~~~~~~~~ ~~~~~~~~~~ ~~~~~~~~~~

TetM *Mho* 6892 ~~~~~~~~~~ ~~~~~~~~~~ ~~~~~~~~~~ ~~~~~~~~~~ ~~~~~~~~~~

TetM *Mho* 5571 ~~~~~~~~~~ ~~~~~~~~~~ ~~~~~~~~~~ ~~~~~~~~~~ ~~~~~~~~~~

TetM *Mho* 6227 ~~~~~~~~~~ ~~~~~~~~~~ ~~~~~~~~~~ ~~~~~~~~~~ ~~~~~~~~~~

TetM *Mho* 6585 ~~~~~~~~~~ ~~~~~~~~~~ ~~~~~~~~~~ ~~~~~~~~~~ ~~~~~~~~~~

TetM *Mho* Sprott ~~~~~~~~~~ ~~~~~~~~~~ ~~~~~~~~~~ ~~~~~~~~~~ ~~~~~~~~~~

TetM *Mho* 2539 ~~~~~~~~~~ ~~~~~~~~~~ ~~~~~~~~~~ ~~~~~~~~~~ ~~~~~~~~~~

....|....| ....|....| ....|....| ....|....| ....|....|

460 470 480 490 500

TetM pMT85-Tet GLSVSPLPLG SGMQYESSVS LGYLNQSFQN AVMEGIRYGC EQGLYGWNVT

TetM *Mho* 5860 ~~~~~~~~~~ ~~~~~~~~~~ ~~~~~~~~~~ ~~~~~~~~~~ ~~~~~~~~~~

TetM *Mho* 6892 ~~~~~~~~~~ ~~~~~~~~~~ ~~~~~~~~~~ ~~~~~~~~~~ ~~~~~~~~~~

TetM *Mho* 5571 ~~~~~~~~~~ ~~~~~~~~~~ ~~~~~~~~~~ ~~~~~~~~~~ ~~~~~~~~~~

TetM *Mho* 6227 ~~~~~~~~~~ ~~~~~~~~~~ ~~~~~~~~~~ ~~~~~~~~~~ ~~~~~~~~~~

TetM *Mho* 6585 ~~~~~~~~~~ ~~~~~~~~~~ ~~~~~~~~~~ ~~~~~~~~~~ ~~~~~~~~~~

TetM *Mho* Sprott ~~~~~~~~~~ ~~~~~~~~~~ ~~~~~~~~~~ ~~~~~~~~~~ ~~~~~~~~~~

TetM *Mho* 2539 ~~~~~~~~~~ ~~~~~~~~~~ ~~~~~~~~~~ ~~~~~~~~~~ ~~~~~~~~~~

....|....| ....|....| ....|....| ....|....| ....|....|

510 520 530 540 550

TetM pMT85-Tet DCKICFKYGL YYSPVSTPAD FRMLAPIVLE QVLKKAGTEL LEPYLSFKIY

TetM *Mho* 5860 ~~~~~~~~~~ ~~~~~~~~~~ ~~~~~~~~~~ ~~~~~~~~~~ ~~~~~~~~~~

TetM *Mho* 6892 ~~~~~~~~~~ ~~~~~~~~~~ ~~~~~~~~~~ ~~~~~~~~~~ ~~~~~~~~~~

TetM *Mho* 5571 ~~~~~~~~~~ ~~~~~~~~~~ ~~~~~~~~~~ ~~~~~~~~~~ ~~~~~~~~~~

TetM *Mho* 6227 ~~~~~~~~~~ ~~~~~~~~~~ ~~~~~~~~~~ ~~~~~~~~~~ ~~~~~~~~~~

TetM *Mho* 6585 ~~~~~~~~~~ ~~~~~~~~~~ ~~~~~~~~~~ ~~~~~~~~~~ ~~~~~~~~~~

TetM *Mho* Sprott ~~~~~~~~~~ ~~~~~~~~~~ ~~~~~~~~~~ ~~~~~~~~~~ ~~~~~~~~~~

TetM *Mho* 2539 ~~~~~~~~~~ ~~~~~~~~~~ ~~~~~~~~~~ ~~~~~~~~~~ ~~~~~~~~~~

....|....| ....|....| ....|....| ....|....| ....|....|

560 570 580 590 600

TetM pMT85-Tet APQEYLSRAY NDAPKYCANI VDTQLKNNEV ILSGEIPARC IQEYRSDLTF

TetM *Mho* 5860 ~~~~~~L~~~ ~~~~~~~~~~ ~~~~~~~~~~ ~~~~~~~~~~ ~~~~~~~~~~

TetM *Mho* 6892 ~~~~~~L~~~ ~~~~~~~~~~ ~~~~~~~~~~ ~~~~~~~~~~ ~~~~~~~~~~

TetM *Mho* 5571 ~~~~~~~~~~ ~~~~~~~~~~ ~~~~~~~~~~ ~~~~~~~~~~ ~~~~~~~~~~

TetM *Mho* 6227 ~~~~~~~~~~ ~~~~~~~~~~ ~~~~~~~~~~ ~~~~~~~~~~ ~~~~~~~~~~

TetM *Mho* 6585 ~~~~~~~~~~ ~~~~~~~~~~ ~~~~~~~~~~ ~~~~~~~~~~ ~~~~~~~~~~

TetM *Mho* Sprott ~~~~~~~~~~ ~~~~~~~~~~ ~~~~~~~~~~ ~~~~~~~~~~ ~~~~~~~~~~

TetM *Mho* 2539 ~~~~~~~~~~ ~~~~~~~~~~ ~~~~~~~~~~ ~~~~~~~~~~ ~~~~~~~~~~

....|....| ....|....| ....|....| ....|....| ....

610 620 630 640

TetM pMT85-Tet FTNGRSVCLT ELKGYHVTTG EPVCQPRRPN SRIDKVRYMF NKIT

TetM *Mho* 5860 ~~~~~~~~~~ ~~~~~~~~~~ ~~~~~~~~~~ ~~~~~~~~~~ ~~~~

TetM *Mho* 6892 ~~~~~~~~~~ ~~~~~~~~~~ ~~~~~~~~~~ ~~~~~~~~~~ ~~~~

TetM *Mho* 5571 ~~~~~~~~~~ ~~~~~~~~~~ ~~~~~~~~~~ ~~~~~~~~~~ ~~~~

TetM *Mho* 6227 ~~~~~~~~~~ ~~~~~~~~~~ ~~~~~~~~~~ ~~~~~~~~~~ ~~~~

TetM *Mho* 6585 ~~~~~~~~~~ ~~~~~~~~~~ ~~~~~~~~~~ ~~~~~~~~~~ ~~~~

TetM *Mho* Sprott ~~~~~~~~~~ ~~~~~~~~~~ ~~~~~~~~~~ ~~~~~~~~~~ ~~~~

TetM *Mho* 2539 ~~~~~~~~~~ ~~~~~~~~~~ ~~~~~~~~~~ ~~~~~~~~~~ ~~~~
